# A computational fluid dynamics modelling of maternal-fetal heat exchange and blood flow in the umbilical cord

**DOI:** 10.1371/journal.pone.0231997

**Published:** 2020-07-28

**Authors:** Dorothea Kasiteropoulou, Anastasia Topalidou, Soo Downe

**Affiliations:** 1 Department of Environmental Sciences, University of Thessaly, Larissa, Greece; 2 Research in Childbirth and Health Unit, School of Community Health and Midwifery, Faculty of Health and Wellbeing, University of Central Lancashire, Preston, United Kingdom; Universite des Sciences et de la Technologie Houari Boumediene, ALGERIA

## Abstract

Human fetal thermoregulation, maternal-fetal heat exchange, and the role of the umbilical cord in these processes are not well understood. Ethical and technical limitations have restricted current knowledge to animal studies, that do not reflect human morphology. Here, we present the first 3-dimensional computational model of the human umbilical cord with finite element analysis, aiming to compute the maternal-fetal heat exchange. By modelling both the umbilical vein and the two umbilical arteries, we found that the coiled geometry of the umbilical artery, in comparison with the primarily straight umbilical vein, affects blood flow parameters such as velocity, pressure, temperature, shear strain rate and static entropy. Specifically, by enhancing the heat transfer coefficient, we have shown that the helical structure of the umbilical arteries plays a vital role in the temperature drop of the blood, along the arterial length from the fetal end to the placental end. This suggests the importance of the umbilical cord structure in maternal-fetal heat exchange and fetal heat loss, opening the way for future research with modified models and scenarios, as the basis for early detection of potential heat-transfer related complications, and/or assurance of fetal wellbeing.

## Introduction

The uterus provides a comparatively stable thermal environment for fetal development. During gestation there is a constant temperature gradient between the mother and the fetus (heat clump) (ΔΤ_mf_), with the fetal temperature being consistently 0.3–0.5°C higher than the maternal core temperature [1–4]. The fetus maintains a higher temperature than the mother, at a constant level of difference, even if the maternal core temperature rises. Although, the fetal thermoregulation is dependent on maternal temperature status and conditions [3, 5–7], the fetus generates heat as a by-product of its metabolism. The heat that is generated by the fetus, is eliminated through the mother [8].

This maternal-fetal thermal gradient, that appears to be the same in all mammals [3–9], including humans [2], allows heat to flow from the warmer fetus to the cooler surroundings and to the mother, in accordance with the first statement of the second law of thermodynamics. The placenta/umbilical cord unit is the main route through which the heat transfer process occurs, and therefore through which this constant thermal equilibrium (the balance between the rate of heat production and heat loss) is achieved [3].

Current knowledge is based on a highly cited study conducted in 1985, which included ten ewe/fetal lamb dyads. In this study, researchers found that 84.5% of the heat that was produced by the fetal lamb exited back to the mother through the umbilical circulation (convective pathway). The remaining 15.5% was dissipated through the fetal skin to the amnion, and then through the uterine wall to the maternal abdomen (conductive pathway) [9]. This and later studies therefore suggested that the umbilical cord (UC), which channels the blood circulation between the fetus and the placenta and vice versa, plays a vital role in this heat transfer process [10, 11]. Several researchers have investigated fetal metabolic processes and heat production, and have assessed the influence of maternal temperature alterations (hyperthermia/fever or hypothermia). However, these studies were all performed in animals (ewes) so there is no direct knowledge about these processes in humans [3–5]. The only related human study (published in 1969) recorded the maternal and fetal temperature, including the temperature of the amniotic fluid and the placenta during Caesarean section. This study did not assess maternal-fetal heat exchange and included no data relating to pregnancy [2].

Animal models are of limited use due to species differences in structure and biochemistry of the fetal/placental unit [12, 13]. The human UC consists of one umbilical vein (UV) that supplies oxygenated and nutrient-rich blood to the fetus and two umbilical arteries (UAs) coiled around it, that transfer the nutrient-depleted blood back to the placenta. These vessels are embedded in Wharton’s jelly and surrounded by a single layer of amnion [10, 11]. In contrast to the human UC, the lamb UC has two UVs and two UAs [14], and its placenta is quite different [15].

Increasing knowledge about the nature and function of the heat-transfer situation of the human fetus could offer potential for understanding aspects of fetal growth, health, and pathology. However, there are obvious ethical and technical limitations to examining this process in-vivo. Consequently, the role of the UC in the human maternal-fetal heat transfer is not well understood.

As a first step in addressing this problem, and to understand for the first time the heat exchange from the fetus to the mother and vice versa, we investigated a 3-dimentional (3D) computational model of the human UC, including the umbilical vein (UV) and both the coiled umbilical arteries (UAs). The effects of the UV and UAs geometry on blood flow properties such as velocity, pressure, temperature, shear strain rate (SSR) and static entropy, were examined. To frame this work, we first undertook a comprehensive scoping review of the existing literature in this area.

## Methods

### Scoping review

The question for the scoping review was to identify what studies exist in relation to the use of 3D computational modelling with finite element analysis (FEA) for the study of the human UC.

The review was undertaken using the methodological framework described by Arksey and O’Malley (2005) [16].

#### Search strategy

The search was conducted in Scopus by two authors (October 2018). A sensitive syntax including a comprehensive list of keywords and medical subject heading (MeSH), was used to ensure that all relevant literature was identified. The search was designed to retrieve all articles combining the concepts of: [TITLE-ABS-KEY] (“umbilical cord”) or (“fetal-placental circulation”) or (“fetoplacental circulation”) and (“computational model*”) or (“computer simulation”) or (computer* models) or (“in silico”) or (“fluid dynamics”) or (“fluid simulation”) or (“numerical study of fluid”). No language restriction was applied. Scopus is a multi-disciplinary database indexing resources in health sciences, engineering, physics etc., which offers 100% coverage of MEDLINE, EMBASE and Compendex. The database includes papers from 1966 [17]. It was considered by the authors as the most suitable, as it contains the majority of the records in the field of our interest.

#### Inclusion/exclusion criteria

Records were not excluded on the grounds of quality, date of study or language [16], as the purpose was to scan the literature and to determine the up to date reported studies and gaps. Only publications presenting the use of computational modelling with FEA of the human UC were included. Publications presenting animal models, not using FEA or not having an abstract or full text available were excluded.

#### Screening process

Two reviewers (AT, DK) screened the identified records by titles and abstract, according to the inclusion and exclusion criteria. Disagreement between reviewers resulted in the article’s inclusion for full-text review. In a second stage, full-text articles were read to identify studies related to our objectives, with any disagreements between reviewers being screened by a third reviewer. The bibliographic reference lists of all included studies were reviewed to identify any additional eligible publications. EndNote X9 software was used to organize the data in the included studies.

#### Charting data and analysis

Two reviewers independently read each included study, extracted relevant data and entered it into two charting tables (Tables 1 and 2). Specifically, we looked at the characteristics of the included studies and recorded them in Table 1. A framework approach was used to identify the basic characteristics of the 3D models only and their parameters (Table 2).

**Table 1:** Summary of papers meeting inclusion criteria.

Author	Year	Country	Stated aim of paper	Type of model	Software	Stated Key Findings (as in original records)
**Kaplan et al [32]**	2010	USA, Israel	*“Develop the first mathematical model of steady blood flow through the coiled structure of an UA”*	3D	ANSYS CFX 10 ANSYS Workbench	*1*. *The driving pressure for a given blood flow rate is increasing as the number of coils in cord structure increases*.
						*2*. *The coiled structure is resulting in interwoven streamlines along the helix and WSS with significant spatial gradients along the cross-sectional perimeter*.
						*3*. *The number of coils does not affect the distribution and levels of WSS (but when the coils are more spread the maximal WSS is significantly smaller*.
						*4*. *Cases with twisted and over coiled (OC) cords seem to yield very large values and gradients of WSS*, *which may place the fetus into high risk of abnormal development*.
**Tejada-Martínez et al [22]**	2011	USA	*“To establish a relationship between the constriction and the appearance of the notch in flow velocity waveforms downstream of the constriction”*	2D	COMSOL (UMFPACK)	*1*. *Notching in envelope flow velocity waveforms (FVW) is not present in flows with less than a 75% constriction*.
						*2*. *Notching disappears as the vortex wave is attenuated at distances downstream of the constriction*.
**Saw et al [33]**	2017	Singapore	*“Characterisation of the umbilical vascular Wall Shear Stress (WSS) environments using clinical ultrasound scans combined with computational simulation*. *Investigation of UA coiling in influencing hemodynamic conditions during the UC bending”*	3D	ANSYS workbench	*1*. *Umbilical vein WSS showed a significant negative correlation with the vessel diameter*, *but UA did not show any correlation*.
						*2*. *Due to the helical geometry of UAs*, *bending of the umbilical cord did not significantly alter the vascular resistance or WSS*, *unlike that in the UVs*.
**Shah et al [20]**	2017	USA	*“The individual and combined effects of umbilical coiling index*, *cord length and arterial diameter on umbilical artery hemodynamics”*	3D	SolidWorks 2016 (Dassault Systemes)	*Specific combinations of umbilical coiling index*, *cord length and arterial diameter yielded pressure and flow drops incompatible with fetal life*
**Saw et al [34]**	2018	Singapore	*“To characterize umbilical vascular WSS environment in normal and intrauterine growth restriction (IUGR) pregnancies*, *and evaluate correlation between WSS and vascular diameter*, *and gestational age”*	3D	ANSYS 18.1 Academic	*Despite having reduced flow rate and vascular sizes*, *IUGR UAs had hemodynamic mechanical stress environments and trends that were similar to those in normal pregnancies*. *This suggested that endothelial dysfunction or abnormal mechanosensing was unlikely to be the cause of small vessels in IUGR umbilical cords*.
**Wilke et al [31]**	2018	Australia	*“A fluid dynamic study of blood flow within the umbilical vessels of the human maternal-fetal circulatory system”*	3D	Oomphlib (open-source finite-element library) and Taylor-Hood elements for simulations	*The presence of vessel helicity dampens extreme pressures within the arterial cycle and may provide another possible evolutionary benefit to the coiled structure of the cord*.

**Table 2:** Geometrical and hemodynamic characteristics from the included studies that used a 3-dimentional model only.

Author	UA, UV, Both	Gestational Age (GA)	Length (L)	Diameter (D)	Pitch	UCI coil/cm	Blood flow	Blood density (ρ) and viscosity (μ)	Blood velocity input (U_in_)	Pressure (P_out_)	Mesh N elements	Specific characteristic
**Kaplan et al [32]**	UA	40 weeks	UA L = 600mm constant	D = 4.0mmconstant	50mm	0.21	Steady, Incompressible, laminar	ρ = 1050kg/m^3^ μ = 0.0033kg*m^-1^*s^-1^	U_in_ = 35cm/s	P_out_ = 0	From 2X10^6^ to 3.7X10^6^	UA had straight and equal sections for the inlet and outlet. Pulsative flow was tested for 1 case compared to steady simulation.
**Saw et al [33]**	UA	32 to 33 weeks	*N/A*	D = 3.0mm constant	5cm	0.2	Steady and unsteady in one case (comparison) Incompressible, laminar	ρ = 1060 kg*/*m^3^ μ = 0.005Pa s	U_in_ = 32.5cm/s	Pulsed Pressure 25mmHg	*1*.2 × 10^6^ to 1.5×10^6^ Appr. 1.2×10^6^ in most cases	Simulations were conducted at peak systolic flow rate, time-averaged flow rate and end diastolic flow rate.
**Shah et al [20]**	UA	40 weeks	*L = 583mm*	D = 4.4mm	N/A	0.4	Steady, Incompressible, laminar	N/A	Constant systolic velocity U = 0.6/m Diastolic velocity U = 0.26 m/s	P_out_ = 20mmHg	N/A	The model does not take into consideration the influence of blood density
**Saw et al [34]**	UA	27 to 39 weeks	*L = 370*.*4mm*	N/A	5cm	0.2	Steady, Incompressible, laminar	ρ = 1060 kg*/*m^3^ μ = 0.005Pa s	Uniform depending on GA	Uniform depending on GA	N/A	Twenty-two normal and 21 IUGR pregnancies were assessed via ultrasound.
**Wilke et al [31]**	UA *UV*	N/A	L = 50cm	0.4 cm (Radius 0.2cm)	50mm	0.3(regular coiled vessels) / 0.2 (nonuniformly coiled vessels) / knots 0.1; 0.2; 0.4 coils/com	Pulsatile-flow compared with steady-flow pressure	ρ = 1060 kg*/*m^3^ μ = 0.004Pa s	Reduced velocity U_red_ = 41	Cross-sectionally average pressure presented	N/A	For UV there is no reference of any calculation or result.

### Computational model

#### Geometry and mesh

**Fig 1:**
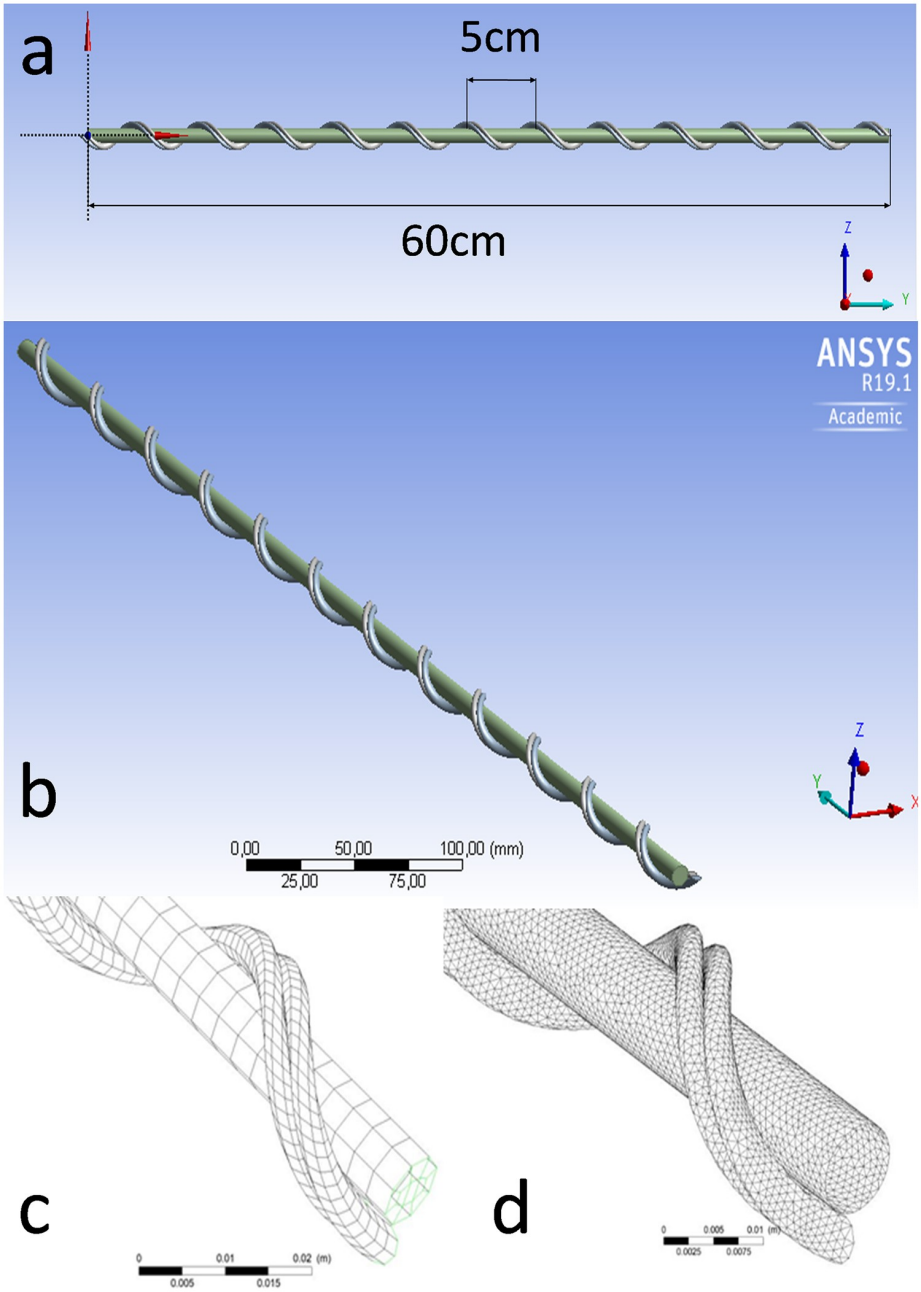
An UC of 60cm total length was modelled (a). The UV was designed as a uniform straight tube, with two uniform UAs twisted over it (b). A sparse and a dense mesh (c) were selected.

ANSYS CFX (R19.1 Academic) was used to study the effects of the UC’s geometry on the blood flow and heat exchange. We developed a model corresponding to 38–40 weeks of gestation. Dimensions and other characteristics were based on information obtained from the literature, related to this gestational age. Although UC at term has considerable variation ranging from 30cm-100cm (<30cm is considered as short and >100cm as long), for the purpose of our model an average length of 60cm was used [11, 18]. The UV diameter was set to 8.3mm and UAs diameter to 4.2mm, both assuming as constant thought the length of the UC [11, 18]. The UC was considered as uniform, with the two UAs twisted counter-clockwise over the UV. UAs had one coil every 5cm along UC’s length (total 12 spirals), which gives an umbilical coiling index (UCI) of 0.2coil/cm [19]. The UV was modelled as a rigid straight pipe and the UAs as rigid helical pipes (Fig 1a and 1b). As, the gelatinous substance of Wharton’s jelly surrounds the UV and UAs, a distance of 0.20 mm between the two surfaces (UV and UAs) was applied. Wharton’s jelly was not of this study’s interest and was not simulated. In order, to achieve numerical convergence and to consume less computer resources and computing time, two mesh designs were used. The percentage differences of both velocity and temperature profiles were calculated less than 5% (Table 3). Initially, a sparse grid was used (Nodes 20,048; Elements 13,750; Hexahedral 13,750) which was then refined to a dense mesh (Nodes 424,216; Elements 1,102,788; Tetrahedral 457,002; Wedges 633,890; Prisms 11,896) (Fig 1c and 1d). A maximum edge length equal to 0.001m and a minimum edge length 0.0001m were chosen to resolve the boundary layers formed near all solid surfaces. For the dense grid the residual Root-Mean-Square (RMS) error values in 1,000 iterations were between 10^−5^–10^−7^ for the mass and momentum components. For the sparse grid the residual RMS error values in 10,000 iterations were between 10^−4^–10^−5^ for the mass and momentum components.

**Table 3:** Velocity and temperature values for UA and UV inputs and outputs for both grids. The two grids showed percentage difference <5%.

		Mesh Type		% difference (<5%)
		Sparse grid	Dense grid	
**Velocity (m/s) mean value**	Artery Input	0.590	0.603	2.179
	Artery Output	0.366	0.355	3.051
	Vein Input	0.304	0.304	0.000
	Vein Output	0.182	0.190	4.354
**Temperature (K) mean value**	Artery Input	310.650	310.648	0.001
	Artery Output	310.386	310.400	0.005
	Vein Input	310.362	310.362	0.000
	Vein Output	310.352	310.362	0.003

#### Mathematical model and computational details

For the purpose of our model, in all simulations the blood flow inside both the UV and UAs was considered as steady, laminar and incompressible. No slip boundary conditions were applied at the vascular walls [20]. During the simulation the mass and momentum conservation equation was used written in the general form

$$\frac{\partial \rho }{\partial t}+\nabla •\left(\rho \vec{U}\right)=0$$ (1)

[In Eq (1), *ρ* is the fluid density and $\vec{U}$ is the velocity vector].

In rectangular Cartesian coordinates, the analytical expression of Eq (1) is expressed as:

$$\frac{\partial \rho }{\partial t}+\frac{\partial (\rho u)}{\partial x}+\frac{\partial (\rho v)}{\partial y}+\frac{\partial (\rho w)}{\partial z}=0$$ (2)

The momentum equation expresses Newton’s second law where the force per unit mass is analysed in forces due to gravity, pressure and consistency. Its general arithmetic expression is as follows:

$$\frac{\partial \rho }{\partial t}+\nabla •\left(\rho \vec{U}⊗\vec{U}\right)=\nabla •\left(-p\delta +\mu \left(\nabla \vec{U}+{\left(\nabla \vec{U}\right)}^{T}\right)\right)$$ (3)

[In Eq (3) *p* is the fluid pressure and $\vec{U}$ is the velocity vector]

Replacing the corresponding terms of the equation gives us the Navier-Stokes equations, which in the case of the rectangular Cartesian coordinates are made respectively:

$$\rho \frac{Du}{Dt}=\rho g_{x}-\frac{\partial p}{\partial x}+\frac{\partial }{\partial x}\left[\mu \left(2\frac{\partial u}{\partial x}-\frac{2}{3}\nabla •\vec{V}\right)\right]+\frac{\partial }{\partial y}\left[\mu \left(\frac{\partial u}{\partial y}+\frac{\partial \text{v}}{\partial x}\right)\right]+\frac{\partial }{\partial z}\left[\mu \left(\frac{\partial w}{\partial x}+\frac{\partial \text{u}}{\partial z}\right)\right]$$ (4)

$$\rho \frac{D\text{v}}{Dt}=\rho g_{y}-\frac{\partial p}{\partial y}+\frac{\partial }{\partial x}\left[\mu \left(\frac{\partial u}{\partial y}+\frac{\partial \text{v}}{\partial x}\right)\right]+\frac{\partial }{\partial y}\left[\mu \left(2\frac{\partial \text{v}}{\partial y}-\frac{2}{3}\nabla •\vec{V}\right)\right]+\frac{\partial }{\partial z}\left[\mu \left(\frac{\partial u}{\partial z}+\frac{\partial \text{w}}{\partial y}\right)\right]$$ (5)

$$\rho \frac{D\text{w}}{Dt}=\rho g_{z}-\frac{\partial p}{\partial z}+\frac{\partial }{\partial x}\left[\mu \left(\frac{\partial w}{\partial x}+\frac{\partial u}{\partial z}\right)\right]+\frac{\partial }{\partial y}\left[\mu \left(\frac{\partial u}{\partial z}+\frac{\partial \text{w}}{\partial y}\right)\right]+\frac{\partial }{\partial y}\left[\mu \left(2\frac{\partial w}{\partial z}-\frac{2}{3}\nabla •\vec{V}\right)\right]$$ (6)

For constant density and shear viscosity the equations are performed:

$$\rho \left(\frac{\partial u}{\partial t}+u\frac{\partial u}{\partial x}+\upsilon \frac{\partial u}{\partial y}+w\frac{\partial u}{\partial z}\right)=\rho g_{x}-\frac{\partial p}{\partial x}+\mu \left(\frac{\partial^{2}u}{\partial x^{2}}+\frac{\partial^{2}u}{\partial y^{2}}+\frac{\partial^{2}u}{\partial z^{2}}\right)$$ (7)

$$\rho \left(\frac{\partial \text{v}}{\partial t}+u\frac{\partial \text{v}}{\partial x}+\upsilon \frac{\partial \text{v}}{\partial y}+w\frac{\partial \text{v}}{\partial z}\right)=\rho g_{y}-\frac{\partial p}{\partial y}+\mu \left(\frac{\partial^{2}\text{v}}{\partial x^{2}}+\frac{\partial^{2}\text{v}}{\partial y^{2}}+\frac{\partial^{2}\text{v}}{\partial z^{2}}\right)$$ (8)

$$\rho \left(\frac{\partial w}{\partial t}+u\frac{\partial w}{\partial x}+\text{v}\frac{\partial w}{\partial y}+w\frac{\partial w}{\partial z}\right)=\rho g_{z}-\frac{\partial p}{\partial z}+\mu \left(\frac{\partial^{2}w}{\partial x^{2}}+\frac{\partial^{2}w}{\partial y^{2}}+\frac{\partial^{2}w}{\partial z^{2}}\right)$$ (9)

As there are limited data related to fetoplacental blood viscosity, the values in our simulation were based on the study from Jouppila et al (1986) [21] who measured the UC blood flow and viscosity 24 hours before delivery and directly after, and the study from Tejada-Martinez et al (2011) [22]. Due to the lack of available data, the flow behaviour index (n) was not included in the computations. The density was considered equal to ρ = 1,056 kg/m^3^ and the viscosity μ = 0.004Ns/m^2^ [21, 22].

The flow was characterised as laminar based on the Reynolds number value, which in our simulation is <2,300 and shows the importance of inertial forces in relation to viscous forces [22]. The Reynolds number is equal to:

$$\text{Re}=\frac{\bar{u}*D*\mu }{\rho }$$ (10)

[In Eq (10), $\bar{u}$ is the mean velocity and *D* is the diameter of the UA and UV]

Specifically, for the input of the UV the Reynolds number is equal to Re = 0.3059(m/s)*0.0083(m)*1,056(kg/m^3^)/0.004(kg*s/m^2^) = 670.288. For the input of the UAs the Reynolds number is Re = 0.6118(m/s)*0.0042(m)*1,056(kg/m^3^)/0.004(kg*s/m^2^) = 678.364.

A constant velocity at the input of each UA was set at *UA*_*in*_ = 61.18cm/s [23] and at the input of the UV at *UV*_*in*_ = 30.59cm/s [11]. For computational purposes, the velocity at the output of the artery was set at *UA*_*out*_ = 35.89cm/s [23] and at the output of the vein at *UV*_*out*_ = 17.95cm/s [11]. The blood flow in the UV has opposite direction from the blood flow in the UAs. The blood flow in the UV has its input at the placental end and the output at the fetal end, while the UAs have their input at the fetal end and the outputs at the placental end.

Knowing that the fetal heat production is 3.5 W*kg^-^’ and that the maternal-fetal temperature difference (ΔΤ_mf_) is 0.5°C, the total heat conductance between the fetus and the mother is calculated as 7 W*kg^-1^* ^ο^C^-1^ [24]. The fetal weight at 39 weeks was set at 3.3kg [25]. The walls were assumed to be smooth with heat flux for the UV walls equal to:

$$Hf_{UV}=\frac{H_{cond}*FW_{39w}*\Delta T_{1}}{A_{w}}$$ (11)

[In Eq (11) *H*_*cond*_ is the heat conductance, *FW*_39*w*_ is the fetal weight at 39 weeks, Δ*T*_1_ is the temperature difference described below and *A*_*w*_ is the wall area).

The heat flux for the UAs walls equals to:

$$Hf_{UA}=\frac{H_{cond}*FW_{39w}*\Delta T_{2}}{A_{w}}$$ (12)

[In Eq (12) *H*_*cond*_ is the heat conductance, *FW*_39*w*_ is the fetal weight at 39 weeks, Δ*T*_2_ is the temperature difference described below and *A*_*w*_ is the wall area).

According to Eqs (11) and (12) the heat flux for the UV and the UAs walls are:

$Hf_{UV}=\frac{7(W/kg^{{}^{\circ}}C)*3.3(kg)*0.088{(}^{{}^{\circ}}C)}{0.0156(m^{2})}=133W/m^{2}$ for the UV wall; and
$Hf_{UA}=\frac{7(W/kg^{{}^{\circ}}C)*3.3(kg)*0.20{(}^{{}^{\circ}}C)}{0.00791(m^{2})}=585W/m^{2}$ for the UA wall;

The temperature difference ΔΤ_1_ was calculated as the difference between the intra-amniotic temperature, which in our case was 37.3°C (310.45K) (fetal temperature 37.5°C-0.2°C) [2], and the temperature in the UV inflow, which was estimated to be equal to *T*_*UVin*_ = 37.212°C(310.362K) (ΔT_1_ = 37.3–37.212 = 0.088°C). The calculation of the UV temperature is described in the next paragraph. The ΔΤ_2_ was calculated as the difference between the intra-amniotic temperature, and the temperature of the UA (ΔT_2_ = 37.3–37.5 = -0.2°C). The temperature at the UAs inflow was set at *T*_*UAin*_ = 37.5°C(310.65K). The outlet temperature for both UV and UAs was left free. The pressure was calculated by the programme. To the best of our knowledge, there is no information available in the literature about the temperature at the input of the UV (placental end). We therefore simulated a maternal vessel to compute the maternal flow into the placenta until its output (input of UV). For simplicity reasons, the model was designed as one straight uniform tube (maternal vessel) that goes through a placental mass of 0.04 thickness [26]. A total pressure of 70mmHg [27] and a total temperature of 37.0°C (maternal core temperature) [2, 28, 29] were set at the input of the maternal vessel. The output velocity was set equal to UV_in_ = 30.59cm/s [11]. The total length of the maternal vessel (tube) was 0.04m. The fetal weight was assumed equal to 3.3kg [25]. The outside temperature of the tube (which is the temperature of the placenta) was set at 0.5°C higher than maternal core temperature [2, 5]. The walls were assumed to be smooth with heat flux equal to:

$$Hf_{MV}=\frac{H_{cond}*FW_{39w}*\Delta T_{2}}{A_{w}}$$ (13)

[In Eq (13) *H*_*cond*_ is the heat conductance, *FW*_39*w*_ is the fetal weight at 39 weeks, Δ*T*_2_ is the the difference between the intra-amniotic temperature and *A*_*w*_ is the wall area]

According to Eq (13) the heat flux is:

$$Hf_{MV}=\frac{7(W/kg^{{}^{\circ}}C)*3.3(kg)*0.50{(}^{{}^{\circ}}C)}{0.00103(m^{2})}=11,213W/m^{2}$$

Based on the abovementioned the output temperature was calculated to be equal to 37.212°C.

## Results

### Scoping review

A total of n = 84 records were identified by the search and screened by titles and abstract. Sixty-seven records were excluded based on the inclusion/exclusion criteria and one for not having an available abstract. Sixteen records were taken forward for full-text review. After excluding 10 records (nine based on inclusion/exclusion criteria and one with no full-text available), six records remained. Five potential additional studies were located through reference checking of included papers, but none met the inclusion criteria, so only the six records located by the electronic search were eligible for inclusion (Table 1). Although the doctoral thesis of Wilke (2016) [30] was relevant to our criteria, it was excluded as it contained similar information to the paper published by the same author [31].

The first use of computational modelling with FEA for the study of the UC was only ten years ago when Kaplan et al. (2010) [32] created the first mathematical model of the coiled UA to assess the pressure and the wall shear stress (WSS). Apart from one study published in 2011 that used a 2D model [22], the other four were published in 2017 and 2018 (Table 1). Most of the 3D published models (n = 5) used similar geometry and blood model, but had differences in their inflow velocity and pressure values (Table 2). The up to date studies were focused either on the investigation of the variations of WSS and blood flow [20, 32–34] or on how knots and notching affect blood flow in UAs [22, 31]. Most of the included studies modeled only the UAs and usually just one UA. Only Wilke et al (2018) [31] described the UV. However, the study was focused on pressure and flow characteristics [31] (Table 2). Based on the above, our study is the first that developed a complete 3D computational model of the UC, including the UV and both the UAs, to investigate heat exchange between the fetus and the mother, including an analysis of the effects of the coiled structure on blood flow velocity, pressure and temperature along the UAs and UV, and their contribution in fetal thermoregulation.

### Simulations

Simulations were conducted to analyse how the UV and UAs geometry affect hemodynamic variables such as blood temperature, blood flow velocity, pressure, SSR, static entropy; and to investigate geometry’s role in the maternal-fetal heat exchange.

#### Temperature and maternal-fetal heat exchange

**Fig 2:**
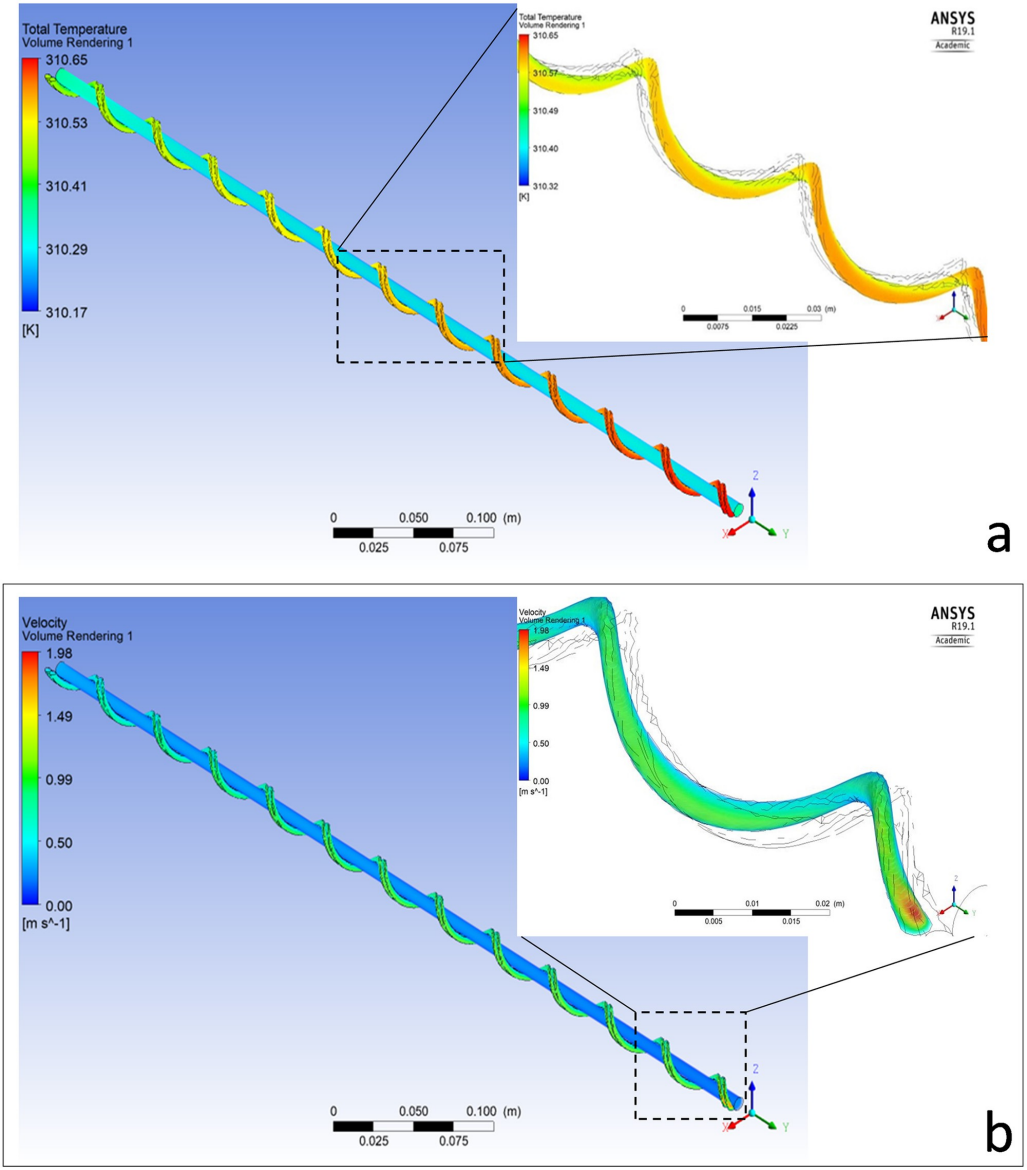
Illustration of the gradual reduction of the temperature as the blood was moving from the input to the output (a). The more helical coils the velocity had to pass the more it reduced towards the output. The reduction of the velocity in the UV was lower (b).

**Fig 3:**
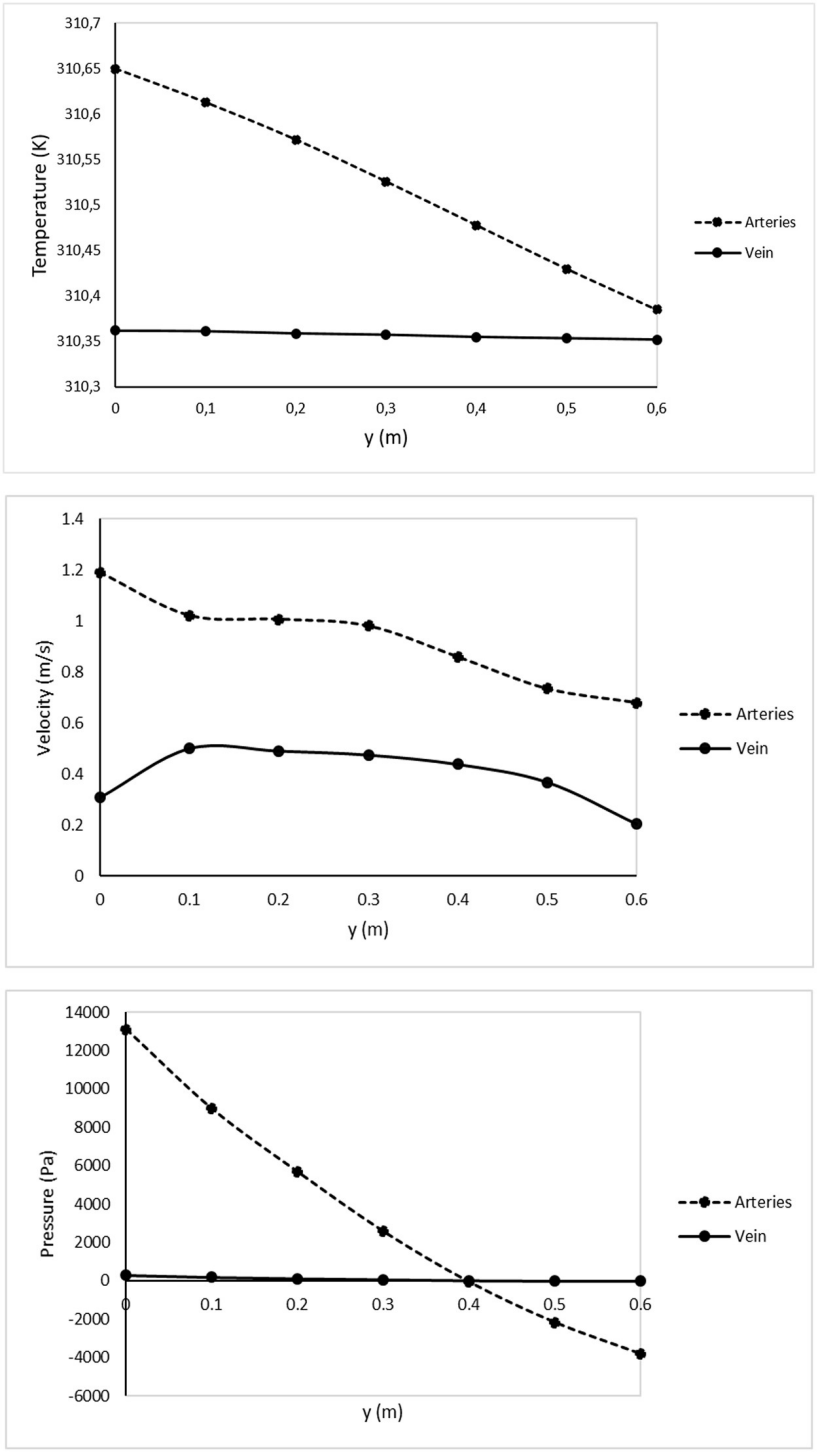
For all the graphs the zero (0) on the x axis is the fetal end (UA input, UV output) and the 0.6m is the placental end (UA output, UV input). The total length of the UC was 0.6m. The temperature drop in the UA, as the blood flowed from the fetus to the placenta is shown in the relevant graph below. It can be observed that the difference in the temperatures of UA and UV at 0m, was almost eliminated at the 0.6m. In comparison, although the reduction of average velocity was noticeable for both UV and UAs, it was not gradual. The pressure drop was significant in the UA, presenting an increase towards the output.

The temperature presented a significant dependence on the helically coiled circular structure of the UAs. The total temperature was 310.65K (37.5°C) at the midpoint of the UA inflow cross-section (fetal end) and dropped to 310.385K (37.235°C) at the midpoint of the outflow (placental end). The gradual reduction of the temperature as the fluid is moving from the one coil to the next (Figs 2a and 3), was more noticeable in the helical cavities, where the resistance to flow is greater. This is because, the fluid in the central part moves outwards by centrifugal forces, and the near the wall fluid is forced inwards. It was observed that as more helical coils the fluid has to pass through, the more its temperature decreases (Figs 2a and 3).

In contrast, the UV fluid temperature remained almost constant. Specifically, the temperature at the midpoint of the UV inflow cross-section (UV_in_ placental end) was 310.362K (37.212°C) and at the midpoint of the outflow cross-section (UV_out_ fetal end) was 310.352K (37.202°C) (Fig 3).

Regarding maternal-fetal heat exchange, the above results showed that the amount of the heat that is supplied by the mother to the fetus, via the placenta, and following the umbilical cord is almost consistent with the amount of heat received by the fetus (UV_in_-UV_out_ = 37.212°C-37.202°C = 0.01°C). On the other hand, the heat that leaves fetus and is transferred to maternal circulation through placenta, showed a significant reduction (UA_in_-UA_out_ = 37.5°C-37.235°C = 0.265°C). This reduction resulted in almost equal temperatures between the UAs and the UV in the placental end (UA temperature = 37.235°C and UV temperature = 37.212°C; ΔT_UAout-UVin_ = 0.023°C) (Fig 3).

#### Flow velocity

The area average velocity was calculated at the midsection of the UAs and the UV. The average velocity presented a reduction in its values moving from the inputs of the UAs and UV towards their outputs (Fig 2b). Higher reduction was observed in the UAs (reduced from 1.189m/s at the input to 0.679m/s at the output), compared to the UV (reduced from 0.306m/s at the input to 0.202m/s at the output). This was due to the UAs coiled shape which forces a reduction in velocity. Also, according to the no slip condition the velocity was zero on the walls (which proofed the proper operation of the model) and had its maximum near the centre of both UAs and the UV. Fig 3 shows the velocity drop in both the UAs and the UV.

#### Pressure

Pressure exhibited a similar behaviour to the temperature and the blood flow velocity. Inside the UV, pressure presented a small drop of just 290Pa between the inflow and the outflow [UV_in_-UV_out_ = abs(-10Pa-280Pa)]. In contrast, inside the UAs pressure drop was equal to 16,910.74Pa [UA_in_-UA_out_ = 13,100.30Pa-(-3,810.44Pa)] (see Fig 3). As presented in Fig 3 the pressure drop in each coil is higher at the coils towards the output than the coils that are closer to the input. As described in the Eqs (2) and (9) the velocity and temperature reduction lead to the reduction of pressure.

#### SSR and static entropy

SSR defines the deformation caused by changes in the temperature, moisture content, chemical reactions and external forces. As in our case, there were no external forces and/or moisture content or chemical reactions, the SSR behavior depended on the temperature changes only. The results showed that in the cross-section of the UV the SSR was constant, whereas in the same cross-section of the UAs the SSR was higher along the walls, showing a reduction towards the centerline of the UA. This revealed that the temperature change and the wall heat flux affected mainly the UAs.

Static entropy was related to the microscopic configurations as it was almost uniform inside the UAs, while presenting an increase in the UV towards the centerline of the middle cross-section. Specifically, near the walls of the UV the static entropy was equal to 138K/KgK, while presented an increase at the centerline equal to 140 J/KgK. On the other hand, for the UAs the entropy in the core of the cross-section was equal to the maximum value of 142.46 K/KgK, and showed a slight decrease equal to 142.02 J/KgK only near the tangent to the UV wall of the artery. This result thereby demonstrating that the static entropy in UAs is almost constant. This indicated that microscopic configurations inside the UAs were more important according to the external wall heat flux’s influence and that the UV wall heat flux influence was more pronounced.

## Discussion

The UC is, literally, the lifeline for the fetus, as well as being a means of maternal/fetal neurohormonal communication. However, despite its vital role, understanding of UV’s and UAs’ hemodynamics and heat exchange properties remained limited, due to the ethical and technical inability to directly observe their functions.

Even though umbilical blood flow research started almost two thousand years ago, when Aristotle (350 BC) reported his observations related to cord clamping [35], the first UV blood flow was first measured by a pulsed Doppler in 1984 [36]. The lack of techniques to investigate UC functions antenatally has restricted technical understanding to what can be ethically observed in animal studies [3–5, 9] or to observations of the human fetus during surgical procedures, such as Caesarean section, or directly after birth, and before the umbilical cord is clamped [2]. Both methods have their limitations. Most current knowledge is based on studies published in the 1980s [5, 6, 9]. However, over the last two decades improved imaging techniques have provided more information related to UC’s structure, functions and hemodynamics [10, 22, 33]. Furthermore, the increase in transdisciplinary collaborations between engineers and clinicians and the advances in FEA during the last ten years [22, 31–34], have resulted in the first computational models of the UC. These models are still limited, as they have generally been focused on the study of a single UA only, examining mainly the effects of the coiled structure on the pressure gradients and the influence of knots in the blood flow [20, 22, 31–34].

As shown from our extensive literature search there is no existing 3D computational fluid dynamics model to date that has included the role of the UC structure in the heat exchange between the mother and the fetus. Although the importance of fetal thermoregulation and of a thermally stable intrauterine environment is well demonstrated [3, 4, 9], there are limited studies investigating the exact route and amount of heat loss from the fetus to the mother.

As stated in the introduction, up to date knowledge is based on studies performed in animals. During the late 1960’ it was believed that the overall heat produced by the fetus was exited by the UC and placenta, back to the maternal circulation [37, 38]. This belief shifted in 1985 based on the study by Gilbert et al [9]. This ewe/fetal lamb dyads study led to the claim that fetal heat loss is accomplished by two avenues: a) heat exchange via the UC blood flow (84,5%), and b) through the fetal skin to the amnion and subsequently to the maternal wall (15.5%). This claim has become the basis of many textbooks in the field [39, 40].

To overcome *in-vitro* and *ex-vitro* limitations, we used an FEA computational modelling for the first time to investigate heat exchange, via the umbilical circulation route. In contrast to the above claims, our findings showed that the amount of heat that is produced by the fetus (specifically this temperature differential-heat clump) does not reach the placental circulation and therefore does not go back to maternal circulation [9]. Our model also demonstrates new knowledge, that the UA’s coiled structure enhances the heat transfer coefficient, accommodating a larger heat exchange surface in a given volume, in comparison with the straight structure of the UV. In our model this resulted in a temperature drop of 0.265°C between the fetal and the placental end of the UA. This suggests that a significant amount of heat is ‘absorbed’ by the UA, resulting in an almost equal UA outflow and UV input temperature (ΔT_UAout-UVin_ = 0.023°C) showing a ‘thermal symmetry’ on the placental end. This finding highlights the importance of the UAs structure and geometry and that parameters related to them, such as the length or the UCI, can play a vital role in the maternal-fetal heat exchange and therefore in fetal thermoregulation. On the other hand, the predominantly straight UV ensures that the amount of heat that flows from the mother to the fetus will reach the fetus almost unchanged.

Velocity and pressure showed a similar behaviour. This can be attributed to the fact that the fluid atoms (blood) are trapped inside the helical curves, which leads to a faster and higher reduction of stream velocities in UAs, in comparison with UV (no curves). The helices act as a friction factor resulting in the reduction of thermal velocities [41, 42]. As presented in Fig 3 the encounter of more coils leads to a gradual reduction in velocity, pressure and temperature. Therefore, the length of the UC and the number of coils are crucial factors.

The difference in pressure drop between helical and the straight tubes has been known since 1908 [43]; and in industry the advantages of helically coiled-tubes, as heat exchangers, are well acknowledged [44, 45]. However, the helical structure of the UC in relation to heat exchange has never been investigated before to our knowledge. In many engineering applications helical tubes are used as the fluid stream in the outer layer flows faster that the fluid stream in the inner layer, resulting in a velocity difference. This difference creates secondary flow by which the heat transfer is increased. Yet, UC is a challenging organ as many parameters that are significant for its modelling remain unknown or are not well-investigated. In addition, the detail of the UC structure differs between almost every pregnancy, in terms of length, diameter and number of coils. Future research could include models of different lengths and diameters, as well as UCIs, in order to understand the influence of these parameters in heat exchange and if there are any thresholds (number of coils or cord length) under or above which this thermal equilibrium is disturbed. In addition, as many UCs have non-uniform coiling, presenting localised regions of hypercoiling or hypocoiling [30], the effect of cord non-uniformities on the blood flow and heat exchange should also investigated. In the future, these findings and potential thresholds could be compared to retrospective clinical data and pregnancy outcomes to create a mechanism of early detection of potential complications, or reassurance of likely health and optimal growth and development for the fetus. Finally, computational modelling of several variations like cord knots, single UA, as well as simulation of hyperthermic and hypothermic conditions would give a deeper insight of the UC effects and hemodynamics.

The direct comparison of our results with the five studies, obtained from our literature search (Table 2) is not applicable due to significant differences in methodologies, geometries and characteristics. Most of the existing studies were focused on the characterisation of the WSS [20, 32, 34], while the scope of our study was to investigate maternal-fetal heat transfer. Velocity profiles of our study revealed similar behaviour with the results presented by other studies where high velocity, which was observed near the centre, skewed towards the outside wall [31–34]. The studies that investigated pressure gradient [31–34] presented results that were in qualitative agreement with those from our study. Apart from the gradual drop of the pressure in the UA, Kaplan et al (2010) [32] showed the driving pressure for a given blood flow is gradually increased with the number of coils (2960Pa for 10 coils) compared with a straight tube (1548Pa). Shah et al (2017) [20] stated that the pressure gradient showed a significant increase for shorted cords (~206mmHg = 27,464Pa), especially when they were hypercoiled (~340mmHg = 45,329Pa). These findings showed the high variability of the pressure gradient that is required to drive blood flow through a coiled structure like the UAs.

There are some limitations in our study. Our model assumed a steady blood flow through the umbilicus. Although it is known that arterial flow is pulsatile [46], the helical structure of the UA results in a more complex flow than the simple Poiseuille flow [47]. In addition, knowing that a theoretical solution to compute flow parameters in helical tubes is still under discussion [47] and that a steady flow simulation in UA can represent an average pattern of the pulsatile flow [32], the steady flow was considered as an acceptable solution which can provide average results, with less computational time and power. Furthermore, the ratio of systolic to diastolic blood velocity, which is described by the pulsatile blood velocity waveforms, differs between a healthy fetus and fetus with growth restrictions, congenital anomalies and other conditions [46]. A future model assessing the heat exchange in fetuses with different conditions, using a condition dependent pulsatile flow would be highly relevant to clinical practice. A second limitation of our study was the modelling of the UAs as rigid coiled pipes, due to the pulsatile flow and systolic/diastolic cycles. Although the UAs are flexible, there is limited knowledge related to their mechanical behavior and stress-strain definition [48]. To model the UV as rigid is considered to be accurate as UV has no pulse pressure [49]. Finally, as we could not find any information regarding the temperature of the UV input, we had to use a simplified computation to calculate this value. The computation and simulation of this structure (maternal vessels, intervillous space, placental end) was outside of the scope of this study. A separate study of a more representative model is needed.

## Conclusion

In conclusion, we developed a 3D computational model for the blood flow in both UAs and UV, to determine the heat exchange between the fetus and the mother in an *in-silico* way. Our results showed that temperature, velocity and pressure presented significant decrease in their values between the UA input and output, while they were almost constant between the UV input and output. We discovered that the helical geometry of the UA played a vital role in the fetal-maternal heat exchange. The curvature effect of the helical coil (greater heat transfer coefficient) resulted in a temperature drop, as the heat was transferred from the warmer fetal end to the cooler placental end. Consequently, fetal blood reached the placenta (UA output) with a temperature almost equal to the temperature of the UV input. This proved that the amount of heat produced by the fetus does not exit back to the mother, and that the UC plays a significant role in fetal thermoregulation. Further computational models of the above-mentioned variations would provide a more complete insight into UC hemodynamics and fetal-maternal heat exchange.
